# Decreased Resting-State Interhemispheric Functional Connectivity in Parkinson's Disease

**DOI:** 10.1155/2015/692684

**Published:** 2015-06-09

**Authors:** ChunYan Luo, XiaoYan Guo, Wei Song, Bi Zhao, Bei Cao, Jing Yang, QiYong Gong, Hui-Fang Shang

**Affiliations:** ^1^Department of Neurology, West China Hospital, Sichuan University, Chengdu, Sichuan 610041, China; ^2^Huaxi MR Research Center (HMRRC), Department of Radiology, West China Hospital, Sichuan University, No. 37 Guo Xue Xiang Chengdu, Sichuan 610041, China

## Abstract

*Background*. Abnormalities in white matter integrity and specific functional network alterations have been increasingly reported in patients with Parkinson's disease (PD). However, little is known about the inter-hemispheric interaction in PD. *Methods*. Fifty-one drug naive patients with PD and 51 age- and gender-matched healthy subjects underwent resting-state functional magnetic resonance imaging (rs-fMRI) scans. We compared the inter-hemispheric resting-state functional connectivity between patients with PD and healthy controls, using the voxel-mirrored homotopic connectivity (VMHC) approach. Then, we correlated the results from VMHC and clinical features in PD patients. *Results*. Relative to healthy subject, patients exhibited significantly lower VMHC in putamen and cortical regions associated with sensory processing and motor control (involving sensorimotor and supramarginal cortex), which have been verified to play a critical role in PD. In addition, there were inverse relationships between the UPDRS motor scores and VMHC in the sensorimotor, and between the illness duration and VMHC in the supramarginal gyrus in PD patients. *Conclusions*. Our results suggest that the functional coordination between homotopic brain regions is impaired in PD patients, extending previous notions about the disconnection of corticostriatal circuit by providing new evidence supporting a disturbance in inter-hemispheric connections in PD.

## 1. Introduction

Parkinson's disease (PD), the second most common neurodegenerative disease worldwide, is characterized by cardinal motor symptoms including tremor, rigidity, bradykinesia, and postural instability. It has been suggested that some motor symptoms in PD might result from impaired sensorimotor integration, in which both deficient afferent external information and high-order cognitive process might play an important role [1, 2]. Some progressive impairment in PD might be a reflection of alterations in integrity of distributed brain networks with resultant reduced information integration capacity between brain regions.

Previous studies have demonstrated that human with sectioned corpus callosum had deficits in the sensory, motor, and cognitive processing [3–6], highlighting the importance of inter-hemispheric coordination to human behaviors. Inter-hemispheric coordination especially is needed for the execution of complex tasks [7, 8]. However, the inter-hemispheric coordination in PD is still an unexplored field. PD is usually of unilateral onset, providing evidence of inter-hemispheric dissociations and an imbalance between activity of the left and right hemisphere [9]. Abnormalities in the corpus callosum and widely impaired white matter integrity in the frontal, temporal, and parietal lobes have been reported in PD patients, which may affect inter-hemispheric functional coordination [10–12]. In addition, preliminary evidence from electroencephalography (EEG) study has suggested impaired inter-hemispheric coordination in patients with PD [13]. Given the importance of bihemispheric coordination for sensory, motor and cognitive processing, and the core motor symptoms in PD, it is reasonable to expect that inter-hemispheric interaction deficits played a key role in the pathophysiology of PD. Therefore, it would be meaningful to examine the inter-hemispheric coordination in PD.

Resting-state fMRI (rs-fMRI), which captures the patterns of coherent spontaneous fluctuations of blood oxygen level dependent (BOLD) signals [14] during rest, can be used to measure the inter-hemispheric coordination. Functional homotopy, defined as the high degree of synchrony in spontaneous activity between geometrically corresponding inter-hemispheric regions, has been suggested to be a key characteristic of the brain's intrinsic functional architecture [15, 16]. Thus, homotopic resting-state functional connectivity (RSFC) may be a sensitive index for detecting the PD-related inter-hemispheric coordination alterations. Here, we examined homotopic RSFC in patients PD using a recently validated approach named ‘‘voxel-mirrored homotopic connectivity (VMHC) [17].” Different strengths of VMHC between different symmetric regions could represent different characteristics of hemispheric specialization in the information processing, sensory integration, and motor coordination [16]. Using the VMHC method, abnormal homotopic RSFC has been demonstrated in schizophrenia [18], autism [19], depression [20, 21], and cocaine addiction [22].

In neurodegenerative diseases, as a diseases process evokes a cascade of pathophysiologic changes, it is important to examine an early stage of the disease that is minimally affected by other confounding factors such as treatment. To minimize the effect of such confounding factors, only drug naive patients with PD were recruited in the present study. Given the extensive evidence of functional disconnections and asymmetry in PD, we hypothesized that an impairment of inter-hemispheric functional coordination may be involved in the pathogenesis of PD, which would be reflected as reduced homotopic RSFC in PD patients. In addition, the values of VMHC might be correlated with the severity of PD symptoms. The greater symptom severity will be associated with lower VMHC values.

## 2. Methods

### 2.1. Participant

Patients with PD were recruited from Movement Disorders Outpatient Clinic of West China Hospital of Sichuan University from January 2010 to February 2012. All PD patients were diagnosed based on the UK PD Society Brain Bank Clinical Diagnostic Criteria. Patients with secondary Parkinsonism and Parkinson-plus syndrome were excluded from this study. At the inclusion to the study, all the patients should have never been treated with anti-Parkinson medications at the initial visit. Patients were excluded if they had (1) moderate-severe head tremor; (2) H-Y stage ≥ 3; (3) disease duration ≥ 4 years; (4) a history of head injury, stroke, or other neurological diseases; (5) dementia; (6) any disorder that interfered with the assessment of the manifestation of PD. In addition, patients with poor response to dopaminergic medication or emergence of non-Parkinsonism symptoms during follow-up period (rang from 12 months to 36 months) will be excluded from the study. Finally, 51 PD patients were included in the study. Most of those subjects are from the same cohort that we used in another recently published work [23]. Functional images were also acquired at initial visit of these patients. The demographic features and clinical data, including age, age of onset, gender, diagnostic delay, and disease duration were collected using a standard questionnaire by a movement disorder specialist during face-to-face interviews at the initial visit. The Unified PD Rating Scale (UPDRS) part III was used to assess the motor disability, and Hoehn and Yahr (H&Y) stage was used to evaluate disease severity. Mini-Mental State Exam (MMSE) was used to evaluate cognition. The ratings were performed blinded to the MRI dataset.

Additionally, 51 right-handed healthy control subjects were recruited from local area by poster advertisements. Control subjects will be excluded if they have (1) any neurological illness, as assessed according to clinical evaluations and medical records and (2) organic brain defects on T1 or T2 images. All the controls were matched for age and sex to patients with PD. The demographic and clinical characteristics of the enrolled subjects are summarized in Table 1. The local research ethics committee approved this study, and written informed consent was obtained from all subjects.

### 2.2. MRI Acquisition

MRI was performed on a 3.0 Tesla (T) MR imaging system (Excite; GE, Milwaukee, WI) by using an eight-channel phased-array head coil. High-resolution T1-weighted images were acquired via a volumetric three-dimensional spoiled gradient recall sequence (TR = 8.5 msec, echo time = 3.4 msec, flip angle = 12°, slice thickness = 1 mm). Field of view (240 × 240 mm^2^) was used with an acquisition matrix comprising 256 readings of 128 phase encoding steps that produced 156 contiguous coronal slices, with a slice thickness of 1.0 mm. The final matrix size of T1-weighted images was automatically interpolated in-plane to 512 × 512, which yielded an in-plane resolution of 0.47 × 0.47 mm^2^. MR images sensitive to changes in BOLD signal levels (TR = 2000 msec, echo time = 30 msec, flip angle = 90°) were obtained via a gradient-echo echo-planar imaging sequence (EPI). The slice thickness was 5 mm (no slice gap) with a matrix size of 64 × 64 and a field of view of 240 × 240 mm^2^, resulting in a voxel size of 3.75 × 3.75 × 5 mm^3^. Each brain volume comprised 30 axial slices and one functional run contained 200 image volumes. The fMRI scanning was performed in darkness, and the participants were explicitly instructed to relax and close their eyes and not to fall asleep (confirmed by subjects immediately after the experiment) during the resting-state MR acquisition. Earplugs were used to reduce scanner noise, and head motion was minimized by stabilizing the head with cushions.

### 2.3. Preprocessing of fMRI Data Analysis

R-fMRI data preprocessing was then conducted by SPM8 software package (http://www.fil.ion.ucl.ac.uk/spm/), REST (http://restfmri.net/forum/rest) and Data Processing Assistant for Resting-State fMRI (DPARSF) [24]. Briefly, the preprocessing steps included the following steps: (1) removal of first 10 time points due to allowing for magnetization equilibrium and the subjects' adaptation to the environment; (2) correction for differences in the image acquisition time between slices; (3) six parameter rigid body spatial transformation to correct for head motion during data acquisition; (4) coregistration of the T1 image to the mean EPI scans; (5) grey and white matter segmentation using “New Segment” and spatial normalization of the structural image to a standard template (Montreal Neurological Institute) by DARTEL “normalization”; (6) spatial normalization of the EPI images using the normalization parameters estimated in the previous preprocessing step and resampling to 3 × 3 × 3 mm^3^; (7) spatial smoothing with a 6 mm full width half maximum Gaussian kernel; (8) temporally bandpass filtering (0.01–0.08 Hz) and linearly detrended removal; (9) regressing eight nuisance covariates, including the white matter signal, the cerebral spinal fluid signal, and six head motion parameters, to remove the possible variances from time course of each voxel.

According to the record of head motions within each fMRI run, all participants had less than 1.5 mm maximum displacement in the *x*, *y*, or *z* plane and less than 1.5° of angular rotation about each axis. We also calculated the mean head translation, mean head rotation, and framewise displacement (FD) [25, 26] for each group. Analysis of those head motion parameters did not reveal differences between the control group and the patient group (*P* > 0.05).

### 2.4. Voxel-Mirrored Homotopic Connectivity Computation

VMHC assumes symmetric morphology between hemispheres. To account for differences in the geometric configuration of the cerebral hemispheres, we firstly averaged the normalized T1 images of all subjects to create a mean normalized T1 image. This image was then averaged with its left-right mirrored version to generate a group-specific symmetrical T1 template. After that, the individual T1 images in MNI space were nonlinearly registered to the symmetrical T1 template and those transformations were applied to the above processed functional data. The VMHC computation was performed with software REST. For each participant, the homotopic RSFC was computed as the Pearson correlation coefficient between each voxel's residual time series and that of its symmetrical inter-hemispheric counterpart. Correlation values were then Fisher *z*-transformed to improve the normality. The resultant values were referred to as the VMHC and were applied for the group comparisons.

### 2.5. Statistical Analysis

When appropriate, two-sample *t*-test and Chi-square tests were performed to assess the differences in demographic and clinical data between PD patients and controls. A two-tailed *P* value of 0.05 was deemed significant. To test for regional group differences in VMHC, individual-level VHMC maps were entered into a group-level voxelwise *t*-test. Significant differences of VMHC between PD patients and controls were set at the threshold of voxelwise *P* < 0.001 and cluster level of cluster size >100 voxel and *P* < 0.05 corrected by familywise error (FWE) correction.

Once significant group differences were observed in any brain areas, we further assessed the relationships between these VMHC values and clinical variables (disease duration and UPDRS-III scores). Pearson correlation analyses were performed, and the significance was set at *P* < 0.05 (two-tailed).

## 3. Results

### 3.1. Demographic and Clinical Characteristics

Age, sex, and handedness were not significantly different between the patients group and the healthy control group. Patients were at early stage of PD with mean disease duration of 1.68 ± 1.02 years (defined as the time since symptom onset). The average H&Y stage was 1.82 ± 0.62. The average motor score on the UPDRS was 24.39 ± 11.62. The clinical data of PD patients are shown in Table 1.

### 3.2. Regional Variation in Voxel-Mirrored Homotopic Connectivity

**Figure 1:**
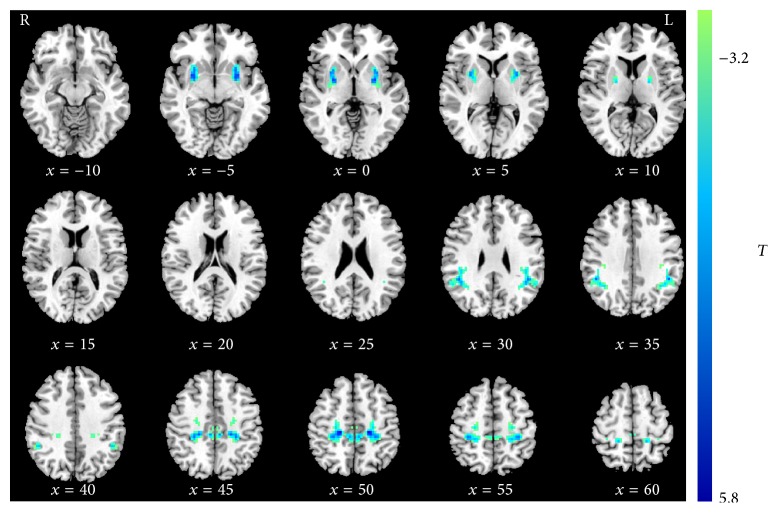
Regions showing significant differences in VMHC between PD patients and healthy controls. Blue colors indicate reduced VMHC in patients compared to the controls. The threshold was set at a corrected *P* < 0.001. The color bar indicates the *T* value from *t*-test between groups. VMHC = voxel-mirrored homotopic connectivity.

Homotopic RSFC was a robust global brain phenomenon, with regional differences in strength (Figure S1 in the Supplementary Material available online at http://dx.doi.org/10.1155/2015/692684), which is consistent with previous work [17]. Group comparisons revealed that patients exhibited lower VMHC than healthy subjects in putamen, sensorimotor cortex (involving precentral, postcentral gyrus and paracentral lobe), and the supramarginal cortex. No region showed greater VMHC in the patient group than in the control group.  Figure 1 and Table 2 showed the group comparisons of VMHC values between patients and healthy subjects.

### 3.3. Correlations between VMHC and Clinical Characteristics

**Figure 2:**
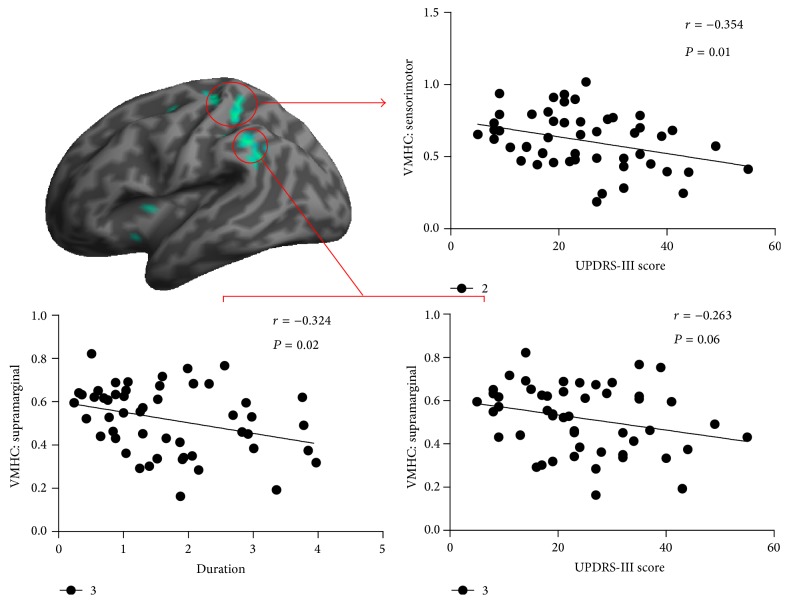
Correlations between the VMHC values and clinical measures in the patient group. VMHC = voxel-mirrored homotopic connectivity; UPDRS = Unified Parkinson's Disease Rating Scale.

The mean VMHC values were extracted in the three regions with significant group differences. Pearson correlations were performed between VMHC and UPDRS motor scores and duration in the patient group. Significantly negative correlation was observed between VMHC in the primary sensorimotor cortex and the UPDRS motor scores (*P* < 0.01) (Figure 2). A trend of negative correlation between VMHC in supramarginal cortex and the UPDRS motor scores was also observed (*P* = 0.06). In addition, VMHC in the supramarginal cortex was also negatively correlated with disease duration (*P* = 0.02) (Figure 2). No significant correlation was found between VMHC in putamen and clinical variables.

## 4. Discussion

Homotopic RSFC is one of the most salient characteristics of the brain's intrinsic functional architecture, and many R-fMRI studies have noted the striking degree of homotopic RSFC [15, 16, 27–29]. Stronger and weaker homotopic RSFC are interpreted as indexing tendencies toward inter-hemispheric coordinated or independent processing, respectively [17]. Here, VMHC was applied for the first time to investigate inter-hemispheric RSFC in PD. Relative to healthy subject, patients exhibited lower VMHC in putamen and cortical regions associated with sensory processing and motor control, which have been verified to play a critical role in the pathology of PD. In addition, there were inverse relationships between the degree of motor disability and VMHC in the sensorimotor regions and between the illness duration and VMHC of the supramarginal gyrus in PD patients.

Parkinson's disease (PD) is characterized by a degeneration of dopaminergic cells in the substantia nigra (SN) pars compacta, which leads to dopamine depletion in the striatum. Putamen is the striatal structure that suffers most from nigro-striatal dopamine depletion. Consistent with its important role in the pathology of PD, we demonstrate lower VMHC value in putamen in patients with PD relative to healthy controls. PD patient typically has a unilateral motor onset, and although the disease becomes bilateral, the initial side commonly remains more afflicted than the later-involved side. This is associated with uneven degeneration of dopaminergic neurons in the nigrostriatal pathway [30]. PD patients with moderate to severe bilateral motor disability still show considerable asymmetry in the putamen and caudate, with relatively reduced DA activity contralateral to the initial motor symptom side [31]. The decreased homotopic RSFC in putamen detected by our study is likely to be associated with the asymmetrical dopamine depletion in putamen.

We also observed a decrease in inter-hemispheric RSFC in some cortical regions, involving primary somatosensory, motor, and supramarginal cortex. Primary sensorimotor cortex is the key structure of sensorimotor circuits, the dysfunction of which has been recognized as a crucial reason for motor difficulties in PD [32]. Abnormal blood oxygenation level dependent (BOLD) fMRI activation and baseline metabolism/perfusion have been reported previously in primary sensorimotor regions in patients with PD [33–37]. Inferior parietal cortex (including supramarginal cortex) is known to be a high-order sensory association area [38, 39] that receives multimdoal sensory afferents and contributes to prception of body scheme and the sensorimtor integration [40, 41]. Disturbed activity and connectivity in this area have also been reported during rest and task-related processes in PD patients [42, 43]. Besides, structural difference in sensorimotor and supramarginal cortex have also been reported in PD [11, 44, 45]. Our results are consistent with the previous finding.

In healthy subjects, the brain motor networks must maintain a dynamic equilibrium during the resting state to integrate bilateral sensory/motor information and be ready to perform a future motor task [46], which requires relatively stringent inter-hemispheric interaction. Accordingly, sensory/motor regions have been demonstrated to exhibited stronger homotopic RSFC than hemispherically specialized frontal and parietal association cortex [16] and increased homotopic RSFC with developmental maturation [17]. Generally, it can be more efficient for the two hemispheres to interact than for one hemisphere to perform all of the processing [47]. If the inter-hemispheric interaction between sensorimotor cortex is disrupted in the resting state, it might lead to deficient inter-hemispheric cooperation, lack of ability to handle complex tasks, and disturbances of sensory processing, and sensorimotor integration, thus contributing to motor impairment. Supporting this, one rs-fMRI study [48] demonstrates that loss and recovery of sensorimotor function decline were paralleled by deterioration and subsequent retrieval of inter-hemispheric functional connectivity within the sensorimotor system in poststroke patients. Consistent with the previous study, our study found a negative correlation between UPDRS motor scores and VMHC in the primary sensorimotor and supramarginal cortex, suggesting deficient inter-hemispheric interaction as a potential mechanism for motor impairment in PD.

A negative correlation was also found between the disease duration and VMHC in the supramarginal cortex, suggesting that this region may have a role in the chronicity of PD. In accordance with our study, a previously published study found an inverse correlation between the cortical thinning of bilateral supramarginal gyrus and illness duration/UPDRS motor score in PD patients [45]. Given that all patients in this study were in early stage with relatively short disease durations, this finding needs to be confirmed in patients with longer illness duration.

It is of interest to speculate on the potential underlying mechanisms of the VMHC deficits which we have demonstrated. They could be related to widespread white matter integrity abnormalities observed in PD. Particularly, deficits in white matter integrity of corpus callosum, the major white matter tract connecting homologous regions of the left and right hemisphere, could disrupt the synchrony between homotopically connected regions. Although the callosum is the largest conduit for information transfer and coordination between the hemispheres, alternative pathways (e.g., subcortical) exist. Homotopic regions with few monosynaptic callosal connections also demonstrated strong resting-state FC [49, 50]. Even studies on split-brain patients have found that a normal complement of resting-state networks and intact functional coupling between hemispheres can emerge in the absence of the corpus callosum [51]. Thus, though with few callosal connections, putamen exhibited decreased inter-hemispheric RSFC in PD which is congruent with the above findings. Anotherexplanation, not mutually exclusive, is that asymmetrical dysregulation of the striatum may in turn lead to further asymmetrical dysfunction of neural circuits that include the basal ganglia and cortical areas. The neurochemical alteration in the basal ganglia impairs neuronal processing and could propagate, through the dense corticostriatal connections, to altered activity in cortical regions. Therefore, the VMHC deficits in sensorimotor regions and putamen could both be related to asymmetrical dopamine depletion in putamen. Further studies are needed to elucidate the mechanism underlying the VMHC alterations in PD.

Several other limitations should be noted. First, there are existing asymmetries in cortical structure. We attempted to mitigate these issues by using a symmetric template. However, the effects of methodological symmetry could not be completely eliminated. Also, the cross-sectional design might limit the interpretations of our results. Whether these regions with abnormal VMHC change dynamically needs to be explored in further longitudinal study. Finally, we recruited only drug naïve patients to reduce effects of confounding factors such as treatment. For the same reason, the present findings might be compounded by a selection bias since drug naive subjects are generally in their early stage.

In summary, we found reduced inter-hemispheric functional connectivity in the key regions of corticostriatal circuit in patients with PD during resting state. This finding extends previous notions about the disconnection of corticostriatal circuit in PD by providing new evidence supporting a disturbance in inter-hemispheric connections in PD. Furthermore, the inverse relations between the motor ability and VMHC of the sensorimotor regions observed in our patients suggest potential clinical implication of VMHC measure for PD.

## Supplementary Material

The supplementary material descries the homotopic RSFC in each group. One-sample tests on the individual VMHC values in each group were performed. Homotopic RSFC was a robust global brain phenomenon, with regional differences in strength. Robust homotopic connectivity was observed in visual, motor, and somatosensory areas, as well as subcortical regions (basal ganglia, thalamus).

## Figures and Tables

**Table 1:** Demographic and clinical characteristics for Parkinson's disease.

	PD (*N* = 51)	Control (*N* = 51)
Age (years)	52.83 ± 8.68	52.24 ± 8.66
Handedness for writing (R : L)	51 : 0	51 : 0
Gender (female : male)	24 : 27	24 : 27
Disease duration (years)	1.68 ± 1.02	—
H & Y stage	1.82 ± 0.62	—
UPDRS scores		
Part I—nM-EDL	2.28 ± 2.15	—
Part II—M-EDL	7.96 ± 4.45	—
Part III—motor examination	24.39 ± 11.62	—
Part IV—motor complications	0	—
Total UPDRS scores	34.43 ± 16.60	—
MMSE scores	27.67 ± 2.67	—

Data are presented as mean ± SD. H & Y = Hoehn & Yahr staging; MMSE = Mini-Mental State Exam; UPDRS = Unified Parkinson's Disease Rating Scale; nM-EDL: Nonmotor Aspects of Experiences of Daily Living; M-EDL: Motor Aspects of Experiences of Daily Living.

**Table 2:** Regions showing significant differences in VMHC between PD patients and healthy controls.

Cluster location	Peak MNI coordinates			Cluster voxels	Peak *T*
	*X*	*Y*	*Z*		
Putamen	±27	−3	−3	141	5.8
Sensorimotor cortex	±18	−27	48	235	5.44
Supramarginal gyrus	±48	−45	33	104	4.77
