# Supplementary material for: Decreased Resting-State Interhemispheric Functional Connectivity in Parkinson's Disease
Source: Biomed Res Int. 2015 Jun 9;2015:692684. doi: 10.1155/2015/692684 (PMC4477209; doi:10.1155/2015/692684)
Supplement: Supplementary file 1 — The supplementary material descries the homotopic RSFC in each group. One-sample tests on the individual VMHC values in each group were performed. Homotopic RSFC was a robust global brain phenomenon, with regional differences in strength. Robust homotopic connectivity was observed in visual, motor, and somatosensory areas, as well as subcortical regions (basal ganglia, thalamus). [file 692684.f1.docx]

**Voxel-Mirrored Homotopic Connectivity.**

One-sample tests on the individual VMHC values in each group were performed. Homotopic RSFC was a robust global brain phenomenon, with regional differences in strength (Figure S). Robust homotopic connectivity was observed in visual, motor, and somatosensory areas, as well as subcortical regions (basal ganglia, thalamus). This is consistent with previous work[1].


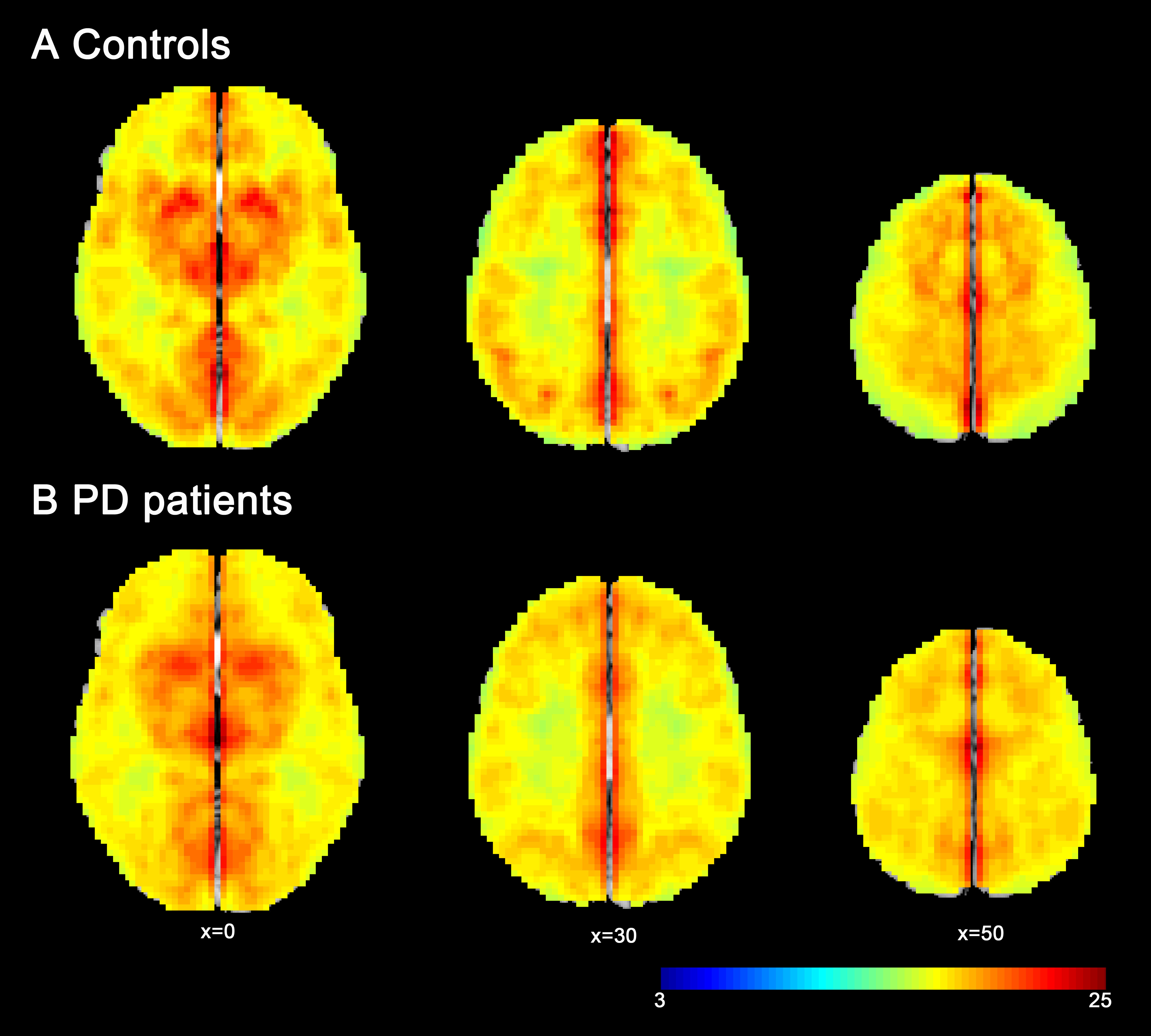


Figure S1: Whole-brain homotopic RSFC pattern. cCorrection for multiple comparisons was performed based on Gaussian random field theory (minimum Z >3.2; cluster level, p < 0.05, corrected).

1. Zuo XN, Kelly C, Di Martino A, Mennes M, Margulies DS, Bangaru S, Grzadzinski R, Evans AC, Zang YF, Castellanos FX *et al*: **Growing together and growing apart: regional and sex differences in the lifespan developmental trajectories of functional homotopy**. *J Neurosci* 2010, **30**(45):15034-15043.
